# Saving Can Save from Death Anxiety: Mortality Salience and Financial Decision-Making

**DOI:** 10.1371/journal.pone.0079407

**Published:** 2013-11-14

**Authors:** Tomasz Zaleskiewicz, Agata Gasiorowska, Pelin Kesebir

**Affiliations:** 1 Faculty in Wroclaw, University of Social Sciences and Humanities, Wroclaw, Poland; 2 University of Colorado at Colorado Springs, Colorado Springs, Colorado, United States of America; George Mason University/Krasnow Institute for Advanced Study, United States of America

## Abstract

Four studies tested the idea that saving money can buffer death anxiety and constitute a more effective buffer than spending money. Saving can relieve future-related anxiety and provide people with a sense of control over their fate, thereby rendering death thoughts less threatening. Study 1 found that participants primed with both saving and spending reported lower death fear than controls. Saving primes, however, were associated with significantly lower death fear than spending primes. Study 2 demonstrated that mortality primes increase the attractiveness of more frugal behaviors in save-or-spend dilemmas. Studies 3 and 4 found, in two different cultures (Polish and American), that the activation of death thoughts prompts people to allocate money to saving as opposed to spending. Overall, these studies provided evidence that saving protects from existential anxiety, and probably more so than spending.

## Introduction

People save and spend money for a variety of reasons, which would map on to Maslow’s hierarchy of human needs, with survival at a low level and symbolic self-expression at a high level [1]. Sometimes saving and spending serve instrumental purposes. Money spent on necessities such as food, shelter, clothing, or healthcare enhances the quantity and quality of one’s life. Saving money can serve the same utilitarian functions, by allowing people to meet these needs at a future time and potentially in emergencies. The uses of money are not restricted to rational, instrumental ends, however. As demonstrated repeatedly by consumer psychologists, money and consumption fulfill important symbolic needs and people’s economic decisions are oftentimes driven by psychosocial concerns (e.g., status) rather than tangible, instrumental utility concerns [2], [3], [4], [5]. One example is consumption as a sexual signaling system: Men have been shown to spend money on conspicuous luxuries as a way to convey their desirability to potential mates [6], [7]. On the other hand, rejecting conspicuous consumption or overspending can also be driven by symbolic reasons, such as a desire for spirituality [8].

The main goal of the present paper is to show that saving behavior can similarly serve symbolic psychological functions. Previous research inspired by terror management theory [9] has consistently documented that money and consumption can buffer death anxiety [10], [11]. The intended contribution of the current paper is to demonstrate that not only spending but also saving money can function as an existential buffer and protect people from death anxiety. In the next part of the paper, we present terror management theory, review the existing literature on how conspicuous consumption helps people to cope with fear of death, and introduce the underlying rationale for our claim that saving can buffer death anxiety. Following that, we describe four experiments that reveal saving as a buffer against existential terror, possibly one that is more effective than spending.

### Terror Management Theory

Humans, unlike other animals, are sophisticated enough in their mental abilities to be aware of the fragility of life and the inevitability of ultimate death. Terror management theory (TMT; for recent overviews, see [9], [12]) proposes that this awareness of mortality has the potential to generate paralyzing anxiety and that the management of this potential anxiety is essential for effective functioning. According to the theory, people develop an anxiety buffering system that, as long as it is functional, protects against existential anxiety and provides psychological equanimity. The key ingredients of this anxiety buffer are a sense of meaning, security, value, relatedness, and transcendence. These ingredients are typically found in cultural worldviews, self-esteem, and close personal relationships. Because these psychosocial entities can buffer against death anxiety, people are highly motivated to seek and maintain them and defend them against threats [13], [14].

Since the inception of TMT about 25 years ago, a large body of research has supported hypotheses generated by the theory (see [15] for a review). Accordingly, when thoughts of mortality are activated, people become more invested in their cultural worldview, self-esteem, and close relationships. For example, reminders of mortality increase hostility toward those who threaten one’s cultural worldview [16], [17], [18], the desire to boost one’s self-esteem [19], the identification with the ingroup [20], and commitment to one’s romantic partner [21]. Conversely, when one’s cultural worldview, self-esteem or close personal relationships are threatened, anxiety increases and death-related thoughts become more salient in the consciousness [22]. Boosting self-esteem or validating one’s worldview, on the other hand, decreases anxiety and pushes death-related thoughts further from consciousness [23], [24]. Taken together, the TMT literature reveals that death anxiety plays an important role in various life domains (e.g., religion and spirituality, politics, sex, health behavior) and that it is a fundamental motivational force for the human psyche.

### Consumption and the Fear of Death

Conspicuous consumption and materialism are two common features of contemporary Western culture. People accumulate material possessions in vast excess to what they need to survive, and sometimes even center their lives around the accumulation of wealth, neglecting other values potentially more conducive to happiness [5], [25], [26], [27]. Scholars have long tried to understand the psychological functions served by materialist behavior, and one of the explanations offered has been that people use wealth as a way to secure meaning and transcend death [28], [29], [30], [31]. Recent empirical research has lent support to the notion that money, wealth, and possessions can buffer existential anxiety. Reminders of mortality have been shown to increase the desire for conspicuous consumption and materialism (for overviews, see [32], [33]). In one study, for example, participants induced to think about their mortality reported higher financial expectations for themselves in the future, and expected to spend more money on pleasurable consumption such as clothing, entertainment, and leisure activities [34]. These participants also became greedier and less environmentally sensitive in a forest management game. Other research found that mortality thoughts increase the desire for high-status luxury products like Lexus automobiles and Rolex watches [10]. Relatedly, Rindfleisch, Burroughs and Wong [35] found that the strong connections materialistic individuals form to brands are motivated by existential insecurity. More recently, Zaleskiewicz and colleagues [11] documented in a series of experiments that money itself possesses a strong psychological anxiety buffering meaning. In these studies, people reminded of their mortality overestimated the physical size of money, used higher monetary standards to define a person or family as rich and desired higher compensation for waiving the immediate payment of money, indicating that they attribute higher value to it. Furthermore, physically interacting with money decreased self-reported death fear, directly attesting to its role as an existential anxiety buffer.

Overall, the studies reviewed above on the symbolic nature of consumption suggest that wealth and material objects may provide a sense of security, value, and meaning, which ultimately function to ward off existential anxiety. This well-established role of consumption as an existential buffer, however, brings up equally important and as of yet unexplored questions: Can saving money help people to manage death-related anxiety as does spending money? If it does, does it potentially constitute a more effective anxiety buffer than spending money? The current research program set out to answer these questions. Addressing a gap in the literature, we investigated the role of saving money as a buffer against existential anxiety and compared it directly to the buffering function of spending money. We hypothesized that saving money would shield against death anxiety, given that it can provide people with a sense of security and value–key ingredients of any existential anxiety buffer. In the next section, we elaborate on our rationale to expect saving behavior to buffer existential anxiety.

### Saving and Fear of Death

Saving means refraining from consumption during one period, in favor of possibilities for consumption at a later period. Saving behavior is considered to be influenced by a complex array of factors, including demographic, economic, and psychological ones [36], [37]. On an individual level, it can be the provider of a number of psychologically desirable–and existentially protective– elements, such as a sense of control, self-esteem, hope and progress. We hypothesize that by providing these elements, saving can buffer against existential anxiety and soothe death fears.

Saving, in its essence, is about dealing with the inherent uncertainty of the future and making (or not making) provisions to ensure having some resources in the future. A major reason for why people save money is to have a buffer against the vicissitudes of life, to be prepared for emergencies (e.g., illness, calamity, loss of livelihood) –in short, to be able to expect the unexpected without fear. As a result, saving money is associated with a sense of control over one’s future and the peace of mind that comes from being ready for the proverbial rainy day.

In addition to being a buffer against uncertainty and diminishing apprehension as to what could happen in the future, saving money can also provide people with a sense of freedom and independence. In contrast, failing to save might mean ending up in debt and dependence. People who have a nest egg have choices and chances in their lives that those who do not save are deprived of. Saving thus means having more of the means to shape existence according to one’s desires, if not now, than sometime in the future. A sense of abundant possibilities and hope about the future can be a potent buffer against existential anxiety.

In light of all its desirable associations, being a successful saver of money can be a source of self-esteem. Indeed, saving is regarded by the majority of people as a sensible, wise behavior. For example, a survey conducted in 2012 on a nationally representative sample of Polish citizens [38] found that over 61% of people perceive saving as a reasonable behavior, and only 23% declare that saving does not make sense. Very similar findings were obtained in previous rounds of this research in years 2008–2011. Moreover, this result is not exclusive to Poland: according to the Consumer Attitudes to Saving Survey [39] conducted in 25 countries in 2008, most people appreciate the value of saving for the future. Specifically, around half of the participants in the surveyed countries declared that saving is a key for a comfortable future (54%) and that they would rather “save for tomorrow than live for today” (45%). For American participants, these numbers were even higher: 64% of them reported that saving is a key for a comfortable future, and 58% reported that they would rather save for tomorrow than live for today. These data suggest that saving may be seen as a valuable, important, and desirable behavior within one’s cultural worldview. As a result, it can not only endow people with a sense of self-worth, but also help people manage impressions in socially commendable ways. In support of this idea, research finds that whereas savers generally tend to make their financial position known to friends and relatives, non-savers tend to keep their finances private and be less forthcoming about it [36]. As Maheswaran and Agrawal [40] argue, consumers are motivated to impress others because it enhances their self-worth, which in turn can shield against existential insecurity. Saving as a socially and culturally supported behavior may be similarly capable of providing self-esteem and hence buffer existential anxiety.

As reviewed above, there are a multitude of reasons for expecting saving behavior to buffer death anxiety. Saving can help weaken the insecurities and anxieties born from the uncertainty inherent to the future. It can help people feel competent and capable as the masters of their financial fate. It can increase the sense of possibility and hope, making people anticipate the future with joy as opposed to with dread. Saving involves investing in one’s future self, which we believe generally to be conducive to existential well-being. Finally, given the socially desirable nature of saving in general, it can be a way for people to attain self-worth within their culture. All this reasoning leads us to hypothesize that saving money will buffer death anxiety.

Previous research revealed that consumption, and conspicuous consumption in particular, shields against death anxiety. An intriguing question becomes whether saving money can be as effective a tool in managing death anxiety as consuming money is. Our intuition is that saving money can be an even more effective anxiety buffer than consuming money, by providing more of some of the existentially protective ingredients than consuming money. Conspicuous consumption can endow people with a sense of self-esteem, born from perceptions of being admired and respected in one’s socio-cultural milieu for one’s consumption. Consumption can provide, albeit temporarily, a promise of transformation and reinvention–a new and better self that will emerge from the act of consumption. While these can effectively buffer death anxiety, we contend that saving money possesses more of existentially protective elements, and as such, can be a sturdier anxiety-shielding tool. Perhaps most importantly, saving can reduce the apprehension born from future-related uncertainties and provide a sense of control over one’s life that conspicuous consumption likely cannot. Saving can also be a more effective means of managing impressions than conspicuous consumption in the context of the current world financial crisis, which renders the pointless waste of money an objectionable behavior.

Self-determination theory provides further theoretical support for our hypothesis that saving would be a more effective anxiety buffer than consuming. The theory posits that psychological health and well-being are associated with the satisfaction of three innate psychological needs–competence, autonomy, and relatedness [41]. If the satisfaction of these three basic needs serves to protect against existential anxiety too, we contend that saving would be a more effective anxiety buffer than consumption, as it seems to do a better job of satisfying the needs of competence, autonomy, and relatedness. We have already discussed the various ways in which saving can produce a sense of competence and autonomy. Saving can also promote relatedness, to the extent that it facilitates providing for and helping one’s loved ones. Kasser [42] notes that although thrift–which can be seen as related to saving in certain respects–is not always associated with enhanced well-being, in cases where it successfully satisfies the psychological needs specified by self-determination theory, well-being results. In contrast, a materialistic and consumerist value orientation seems to thwart the satisfaction of the competence, autonomy, and especially relatedness needs, partly explaining its association with diminished personal well-being [43]. Thus, self-determination theory offers another rationale for why we should expect saving to be more successful in soothing existential anxiety than consuming.

The hypotheses presented above are partly supported by research showing that young people who had experienced the death of a close other by cancer tended to favor long-term future over short-term interests when making intertemporal choices [44]. Specifically, the participants with death experience, compared to controls, allocated more money to long-term funds, focused more on future perspectives when making consumer decisions, and preferred activities that were framed as long-term goals. Liu and Aaker [44] bring up terror management theory in their work, but interpret their results mainly as suggesting that death experience, especially at a younger age, increases the salience of a future life course and shifts focus from short-term toward long-term consequences. Not excluding this explanation, we argue that saving can also be a tool for people to deal with existential anxiety. This tool becomes more relevant when people are reminded of their mortality and seek to protect themselves against existential terror.

In the remaining part of this paper, we present results of four experiments that support the argumentation presented above. These studies show that mortality salience motivates decision-makers to save more, and that saving primes diminish participants’ fear of death, to an even higher degree than spending primes.

### Overview of Studies

Study 1 tested whether making thoughts of saving or spending salient, compared to a control condition, would decrease participants’ fear of death. Based on previous research concerning TMT and consumption, and our above-presented reasoning on the existentially protective role of saving, we expected that thoughts of saving would soothe death anxiety– to at least the same degree as thoughts of spending money. Study 2 examined whether reminders of mortality increase the perceived attractiveness of saving, compared to spending, and motivate people to make decisions serving long-term financial goals. Finally, Studies 3 and 4 were designed to test how death thoughts affect people’s decision to allocate money to saving versus spending.

### Ethics Statement

All experiments in the present research were approved by the Ethics Committee of Psychological Research at the University of Social Sciences and Humanities, Faculty in Wroclaw. All subjects in Study 1–3 signed written informed consent prior to participation. For participants who completed the study via the Internet (Study 4), consent was provided by clicking a designated button on-line.

## Study 1

The goal of Study 1 was to examine the effects of activating spending or saving thoughts on participants’ self-reported fear of death. Recent research has revealed that money, wealth, and possessions serve to buffer death anxiety. However, research on the topic typically tested this buffering hypothesis by looking at how people’s spending behavior is affected by mortality reminders [10], [32], [34], [35]. The current study entails a more direct examination of the hypothesis that economic activity can buffer death anxiety. Specifically, it tested whether thoughts of spending and saving money would be associated with lessened death anxiety. At the same time, we also aimed to discern which type of economic activity (i.e., spending vs. saving) would be more effective in soothing death anxiety. We hypothesized that both saving and spending thoughts would reduce death anxiety relative to a control condition, and saving thoughts would be even more effective than spending thoughts.

### Participants

We recruited 139 Polish adults who were employed and had their independent income. Participation in the study was voluntary and participants did not receive any compensation. The experiments were conducted individually using the paper-and-pencil method in the university lab. Nineteen cases were omitted due to missing data, either because the participant did not entirely complete the priming task or the dependent variable. Final analyses were thus performed on data collected from 120 participants (aged 19–67 years, *M* = 30.47, *SD* = 11.82; 89 females). All study materials and interactions with participants were in Polish.

### Design and Materials

Participants were told that they are going to complete a short (5-minute) survey on “imagination and personality” and assigned randomly to one of three conditions: weather (control condition, n = 40), spending (experimental condition 1, n = 40) and saving (experimental condition 2, n = 40). They were asked to imagine and write down five positive outcomes of: (1) having great weather on the next day (in the control condition), (2) getting 10,000 PLN (app. $3,330) and spending it for pleasure and luxuries (in experimental condition 1) or (3) getting 10,000 PLN (app. $3,330) and saving it for the future (in experimental condition 2). After this priming task, all participants completed the brief version of the Positive-Negative Affect Scale (PANAS [45]). The PANAS was added to control for possible mood differences between the three conditions.

Next, participants were asked to complete the fear of death [46] and fear of dentist questionnaires in randomized order. These questionnaires have been used to manipulate mortality salience in earlier TMT studies [13], [18]. Dental pain is oftentimes used as a comparison condition for mortality salience, because it is a highly unpleasant yet not lethal type of pain. In the present study, we assessed fear of dental pain to test our claim that primes of saving and spending would have an impact on the existentially motivated death fear, but not on the non-existential dental fear.

The “fear of death questionnaire” [46] consists of 12 items related to death anxiety (e.g., “I am very much afraid to die”), to which participants respond with ‘yes’ or ‘no’. The “fear of dental pain questionnaire” consists of 12 similarly worded items on dental pain (e.g., “I am very much afraid of dental work”), again with ‘yes’ or ‘no’ as answers. Each response indicating fear of death/dental pain was coded as 1, and responses reflecting the lack of fear were coded as 0.

### Results

Preliminary analyses indicated that participants assigned to the three conditions did not differ among themselves in their positive, *F*(1, 117) = 0.80, *p* = 0.45, or negative affect *F*(1, 117) = 0.72, *p* = 0.49.

To test the research hypothesis, a 2 (type of fear: mortal vs. dental, within-participants factor) by 3 (prime: weather vs. spending vs. saving, between-participants factor) repeated measures ANOVA was conducted. A significant interaction between type of fear and experimental prime was obtained, *F*(2, 117) = 8.34, *p*<0.001, *η^2^* = 0.13 (sphericity assumed). Specifically, we found a significant effect of experimental condition on fear of death, *F*(2, 117) = 11.85, *p*<0.001, *η^2^* = 0.17, but not on fear of dental pain, *F*(2, 117) = 0.44, *p* = 0.65. The descriptive statistics for fears of death and dental pain for the three groups are presented in Table 1.

**Table 1:** Level of death and dental fear in three experimental conditions (standard deviations in parentheses).

Type of fear	Weather	Spending	Saving	Overall
Fear of death	7.08 (2.62)	5.78 (2.09)	4.64 (1.96)	5.83 (2.44)
Fear of dentist	3.89 (2.59)	4.30 (2.97)	4.45 (2.79)	4.21 (2.77)

Further t-tests revealed, in line with our hypotheses, that participants primed with thoughts of spending declared lower fear of death (*M* = 5.78, *SD* = 2.09) than those in the control condition (*M* = 7.08, *SD* = 2.62), *t*(78) = 2.46, *p* = 0.016, Cohen’s *d* = 0.28. Similarly, participants primed with thoughts of saving declared lower fear of death (*M* = 4.64, *SD* = 1.96) than participants in the control condition (*M* = 7.08, *SD* = 2.62), *t*(78) = 4.18, *p*<0.001, Cohen’s *d* = 0.53. Moreover, the difference between the two experimental conditions was also significant, *t*(78) = 2.51, *p* = 0.014, Cohen’s *d* = 0.28, indicating that the saving prime was more effective in soothing death fear than was the spending prime.

A subsequent analysis of covariance (ANCOVA) that controlled for some potentially relevant covariates (gender, age, positive affect, negative affect) confirmed the significance of the experimental main effect on death fear, *F*(1, 113) = 10.92, *p*<0.001, *η^2^* = 0.16. None of the covariates in the model reached statistical significance (*p* = 0.32 for gender, *p* = 0.63 for age, *p* = 0.42 for positive affect, *p* = 0.49 for negative affect).

## Study 2

Study 1 showed that thinking about either saving or spending money reduces fear of death relative to a control condition. Furthermore, saving primes were more effective than spending primes in lowering death anxiety. These findings provided preliminary support for our hypotheses. One limitation of the study, however, was that the participants did not face a “save or spend” dilemma. Saving can often be interpreted in terms of delayed consumption [37]. It is hence possible that the specific saving prime in Study 1 led participants to conceive of saving as accumulating money with the ultimate goal of spending it on pleasure and luxuries in the future. In other words, the saving prime could have been confounded with elements of spending. To address this issue, we conducted a second experiment in which we juxtaposed saving and spending goals, and observed how participants chose between saving and spending under mortality salience.

The main goal of Study 2 thus was to examine how mortality salience affects the way people make save-or-spend decisions. In this experiment, we were not only interested in saving that is explicitly related to a certain purpose (e.g., future purchases and consumption) but also in saving for the sake of saving, when no future goal is specified. If saving money is capable of providing more protection against death anxiety than does spending money, mortality salience should lead people experiencing a “save or spend” dilemma to make more prudent and frugal choices. Accordingly, it was hypothesized that participants in the mortality salience condition would choose frugal behavior more often than participants in the control condition.

### Participants

We recruited 92 Polish adults (aged 19–72 years, *M* = 35.65, *SD* = 15.06; 58 females), who were all employed and had their independent income. Participation in the study was voluntary, and no compensation was offered for participating. The experiments were conducted individually in the university lab using the paper-and-pencil method. All study materials and interactions with participants were in the Polish language.

### Design and Materials

To prime participants with thoughts of mortality, we used a procedure commonly employed in TMT studies (e.g., [13]). Participants were randomly assigned to either a mortality salience (n = 45) or control (n = 47) condition. Following Rosenblatt et al. [18], participants in the mortality salience condition completed the “fear of death questionnaire” [46] described in Study 1. Participants in the control condition, on the other hand, filled out the “fear of dental pain questionnaire”. The aim of these two questionnaires was not to assess level of death or dental fear per se, but to activate thoughts about mortality in the experimental group and thoughts about dental pain in the control group. The questionnaires have thus not been scored.

Following this manipulation, all participants completed the brief version of the Positive-Negative Affect Scale (PANAS; [45]) to allow us to account for possible mood differences between the two conditions. Next, participants solved a crossword puzzle, the purpose of which was to draw attention to another topic and push the recently evoked thoughts about death out of consciousness. Previous research has established that delay and distraction tasks following mortality reminders lead to more robust terror management effects, and that different types of defenses are elicited when thoughts of death are in focal consciousness [15], [47]. Typically, when mortality thoughts are under focal attention, they result in proximal defenses aimed at dealing with mortality concerns in a relatively direct and rational fashion (e.g., by resolving to eat better, to exercise more, to have more regular check-ups etc.). Distal defenses, on the other hand, emerge only when thoughts of mortality have faded to the fringes of consciousness. They attempt to cope with the problem in a more indirect, symbolic manner, such as through worldview defense or self-esteem striving. This distinction is especially important for our purposes, because saving and spending can function as both distal and proximal buffers. Money can be thought of as a proximal buffer, given that it can procure better access to resources promoting physical safety and health. Simultaneously, it can be thought of as a distal buffer, given its symbolic meaning as a provider of existential security and value (see [11]). Our analysis emphasizes the symbolic power of money and its distal existential buffering function. As a result, we included a delay and distraction task following mortality salience, in an attempt to allow the hypothesized distal effects to occur.

The final task in the study aimed to capture the dependent variable and involved making decisions in a save-or-spend dilemma scenario. The task consisted of responding to two simple scenarios: “TV Set” and “Earned Money”, presented to the participants in randomized order.

In the “TV Set” scenario, participants were asked to imagine that they wanted to buy a Sony Bravia 40″ TV that costs 2,500 PLN (app. $833), but they had only 1,500 PLN (app. $500). Given this situation, they were asked to choose one from among three possible courses of action: (1) save money for the next five months and then buy the TV (frugal behavior); (2) use the 1,500 PLN to buy the TV now and finance the remaining amount using an installment plan (moderate behavior); and (3) make the whole purchase with an installment plan and spend the 1,500 PLN on other things right away (spendthrift behavior). It should be noted that in all the options presented above, the decision concerned consumption, but with different financing possibilities.

In the “Earned Money” scenario, participants were asked to imagine that they had earned a sum of 8,000 PLN (app. $2,666) after they worked abroad for half a year, and, now, after coming back home, they were about to decide what to do with this money. They were asked to choose one from three possible courses of action: (1) save the whole amount of money for the future (frugal behavior); (2) save some of the money, and spend the rest for pleasure and luxuries (moderate behavior); and (3) spend the whole amount of money right away for pleasure and luxuries (spendthrift behavior).

### Results

Preliminary analyses indicated that participants assigned to the mortality salience condition and the control condition did not differ among themselves in their positive, *F*(1, 90) = 0.04, *p* = .85, or negative affect, *F*(1, 90) = 0.01, *p* = .91. In other words, the mortality salience effects observed in this study were not mediated by mood.

Chi-square tests revealed significant differences between the mortality salience condition and the experimental condition in the distribution of choices for both the “TV Set” scenario, *χ* ^2^(2, *N* = 92) = 5.92, *p* = .052, and the “Earned Money” scenario, *χ^2^*(2, *N* = 92) = 7.96, *p* = .019. As shown in Table 2, in the mortality salience condition, the frugal option was chosen more frequently, and the spendthrift option was chosen less frequently compared to the control condition for both scenarios. Thus, mortality salience seemed to induce a stronger desire for saving as opposed to spending in a forced choice situation. This provided additional support for our hypothesis that saving behavior can buffer death anxiety and be even more effective at that than spending behavior.

**Table 2:** Percentages of spendthrift, moderate and frugal choices for the “TV set” and “Earned money” scenarios.

Choice	TV set scenario		Earned money scenario	
	control condition	experimental condition	control condition	experimental condition
spendthrift	31.11	21.28	33.33	10.64
moderate	44.44	29.79	62.22	76.60
frugal	24.44	48.94	4.44	12.77

## Study 3

The goal of Study 3, similar to Study 2, was to examine how mortality salience affects people’s decisions in a save-or-spend dilemma. In line with the results of Study 2, we expected that mortality salience would make people experiencing a save-or-spend dilemma less willing to consume and more willing to save. However, in the present experiment the dependent variable was operationalized differently from Study 2. Participants were asked to imagine that they unexpectedly received an amount of 10,000 PLN (app. $3,330) and could divide it among different options, ranging from long-term savings (most frugal option) to spending it on luxury products and pleasure (least frugal option).

In light of our argument that saving has a greater existential anxiety-buffering capacity than consuming, we hypothesized that participants in the mortality salience condition would ascribe more money to savings and less money to consumption than participants in the control condition.

### Participants

We recruited 92 Polish adults, who were all employed and had their independent income. Participation in the study was voluntary, and no compensation was offered to participants. Four participants were excluded from the analysis, because their responses to the dependent variable question did not add up to 10,000 PLN as they were supposed to. The final data analysis was thus conducted on a group of 88 participants (aged 19–72 years, *M* = 35.86, *SD* = 14.96; 56 females). Each participant was tested individually in the university lab and completed paper questionnaires. All study materials and interactions with participants were in Polish.

### Design and Materials

To prime participants with thoughts of their mortality, we used the same procedure as in Study 2. Participants were randomly assigned to either the mortality salience (n = 44) or the control (n = 44) condition. In the mortality salience condition, participants completed a fear of death questionnaire, while those in the control condition filled out the fear of dental pain questionnaire. Following the manipulation, all participants completed the brief version of the Positive-Negative Affect Scale (PANAS; [45]). Next, they solved a crossword puzzle, which was aimed as a distracter. Participants’ final task was to imagine that they got a windfall of 10,000 PLN ($3,330) and had to divide this amount among four options: (1) long-term savings (like a retirement plan or savings account); (2) immediate-access savings (money kept under the mattress); (3) everyday expenses; and (4) luxury, pleasurable consumption and dreams fulfillment. Our main interest was in the total amount of money allocated to saving vs. spending. The options differentiated between long- vs. short-term saving and ordinary vs. extraordinary spending, however, this aimed at making the true nature of the task less obvious to the participants and mask our main interest in how they would partition between saving and spending. Thus, in data analysis, participants’ answers to options 1 and 2 were summed up to form one variable reflecting total money ascribed to saving, and options 3 and 4 were summed up to form another variable reflecting total money ascribed to spending.

### Results

Preliminary analyses indicated that participants assigned to the mortality salience condition and the control condition did not differ in positive, *F*(1, 86) = 0.235, *p* = .63, or negative affect *F*(1, 86) = .002, *p* = .96. The mortality salience effects found in the study were apparently not mediated by mood.

**Figure 1:**
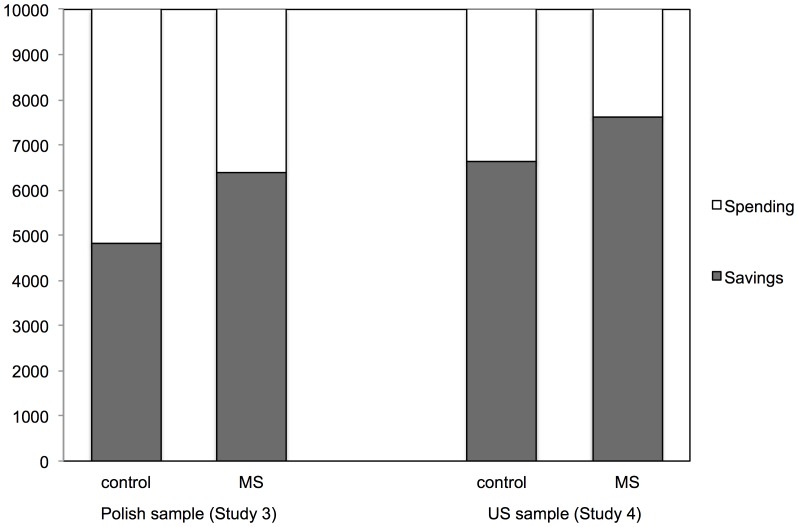
Average sums ascribed to saving and spending in the Polish sample (in PLN) and in the US sample (in $).

Our two dependent variables (money allocated to saving and money allocated to spending) by necessity add up to 10,000 PLN. As a result, the main effects of experimental condition for both variables were the same and also identical to the interaction effect between type of goal and experimental manipulation in a 2 (type of goal: saving vs. spending, within-participants factor) by 2 (manipulation: mortality salience vs. control condition, between-participants factor) repeated measures analysis of variance. So, we report only one set of statistics. As expected, a significant effect was observed, *F*(1, 86) = 7.48, *p* = .008, *η^2^* = .08. Participants in the mortality salience condition reported that they would save more money and spend less money than those in the control condition (see Figure 1). Specifically, participants reminded of dental fear reported that they would allocate similar amounts of money to saving (*M* = 4,818.18 PLN, *SD* = 2,894.63) and to spending (*M* = 5,181.82 PLN, *SD* = 2,984.63), *F*(1, 43) = 0.16, *p* = .69. Participants reminded of the fear of death, on the other hand, reported that they would allocate significantly more money to saving (*M* = 6,386.36 PLN, *SD* = 2,984.63) than to spending (*M = *3,613.64, *SD* = 2,359.67), *F*(1, 43) = 15.19, *p*<.001, *η^2^* = .26.

The analysis of covariance confirmed the significance of the manipulation, *F*(1, 82) = 7.97, *p* = .006, *η^2^* = .089. None of the covariates reached statistical significance (*p* = .13 for gender, *p* = .38 for age, *p* = .09 for positive affect, *p* = .995 for negative affect).

The results from Study 3 were entirely consistent with our hypotheses and the previous two studies. They showed once again that saving plays a symbolic anxiety buffering role in the face of death thoughts, and makes spending a relatively less appealing option.

## Study 4

The objective of Study 4, similar to Study 3, was to examine whether mortality salience would prompt people in a save-or-spend dilemma to save more and spend less. Differently from Study 3 and the remaining studies reported in the article, the present study was conducted on an American sample. We wanted to verify that the observed existential buffering effect of saving was not unique to Poland and could be generalized to another culture. We specifically chose an American sample, because most of the TMT studies, especially those concerning materialism and consumption as death anxiety buffers, have been conducted in the United States. It would be a valuable contribution to demonstrate in the same population that saving too can be a death anxiety buffer. If saving shields against the anxiety born from the uncertainties of the future, and provides people with a sense of control, competence, and self-worth, then it would help Americans to deal with death anxiety as well. Indeed, long-term savings seem to be even more popular and more culturally promoted in the U.S. than in Poland [39], reinforcing our hypothesis that reminders of death would motivate Americans to save more for the future as well.

### Participants

Ninety-nine American participants were recruited from Amazon’s Mechanical Turk marketplace, and completed a brief online survey in exchange for $0.40. Eighteen participants were excluded from the analysis because their responses to the dependent measure did not add up to $10,000 as it was supposed to. The final analysis was thus conducted on a group of 81 participants (aged 18–64 years, *M* = 31.48, *SD* = 9.883; 33 females). All study materials were in English.

### Design and Materials

To prime participants with thoughts of mortality we used a procedure that was different from the one used in Studies 2 and 3, but commonly employed in TMT studies. Participants were randomly assigned to either the mortality salience (n = 43) or the control (n = 38) condition. In the mortality salience condition, they were asked to write three sentences about what they feel when they think about the fact that they will die one day, whereas in the control condition they were asked to write three sentences about what they feel when they think about experiencing intense pain during a visit to the dentist. As in Studies 2 and 3, all participants completed the Positive-Negative Affect Scale (PANAS; [45]) following the manipulation and solved a crossword puzzle. Participants’ final task was identical to the one used in Study 3–namely, they were told that they received a windfall in the amount of $10,000 and were asked to divide the money among four financial options. The amount of $10,000 was comparable in its real value to the 10,000 PLN used with the Polish sample in Study 3, given the average gross income in the two countries. The four financial options, as in Study 3, were: (1) Long-term savings; (2) Savings that are accessible “here and now”; (3) Everyday expenses; and (4) Pleasures, luxuries and dream fulfillment. Similar to Study 3, the responses given by the participants to options 1 and 2 were summed up to form one variable reflecting total money ascribed to saving, and options 3 and 4 were summed up to form a variable reflecting total money allocated to spending.

### Results

Preliminary analyses revealed that participants in the mortality salience and control conditions did not differ in positive affect, *F*(1, 79) = 0.83, *p* = .365, or negative affect, *F*(1, 79) = 0.06, *p* = .81. That is, the mortality salience effects obtained in the study were not mediated by mood.

As in Study 3, our two dependent variables by definition sum up to $10,000 and elicit identical results in a 2 (type of goal: saving vs. spending, within-participants factor) by 2 (manipulation: mortality salience vs. control condition, between-participants factor) repeated measures analysis of variance. We thus again report only one set of statistics. Results yielded a significant effect of manipulation, *F*(1, 79) = 4.89, *p* = 0.03, *η^2^* = 0.058. As the Polish participants in Study 3, American participants in the mortality salience condition reported that they would save more money and spend less money than those in the control condition (see Figure 1). Participants reminded of dental fear ascribed significantly more money to saving (*M* = $6,635, *SD* = 2,471.43) than to spending (*M* = $3,365, *SD* = 2,471.43), *F*(1, 37) = 16.631, *p*<.001, *η^2^* = .31. However, participants reminded of death reported that they would allocate even more money to saving (*M* = $7,622.09, *SD* = 1,476.74) than to spending (*M* = $2,377.91, *SD* = 1,476.74), and this effect was twice as strong as in the control condition, *F*(1, 42) = 135.57, *p*<.001, *η^2^* = .76.

An analysis of covariance that included the potentially relevant variables of gender, age, positive affect and negative affect confirmed the significance of the manipulation, *F*(1, 75) = 5.35, *p* = .024, *η^2^* = .067. None of the covariates reached statistical significance (*p = *.172 for gender, *p* = .50 for age, *p* = .56 for positive affect, *p* = .62 for negative affect).

Though the participants’ money allocation patterns in this study were not fully comparable to the patterns obtained with Polish participants, we replicated our main finding regarding the anxiety buffering function of saving. American participants displayed an even greater interest in saving following mortality thoughts than Polish participants did.

## General Discussion

The main goal of the studies presented in this paper was to show that saving money can serve as a symbolic buffer against existential anxiety. Results of the four experiments revealed, in line with the title of the paper, that saving money can save from the fear of death. To our knowledge, this was the first empirical attempt to demonstrate the symbolic existential power of saving money and also to pit saving against consuming to assess the comparative merit of each in buffering death anxiety. Our first experiment demonstrated that people report lower fear of death when they have been asked to think about either saving or spending money, but not about a control topic. Furthermore, saving primes were more effective in reducing death fear than spending primes. Experiments 2 and 3 revealed that making mortality salient increases the likelihood of choosing frugal options and investing more money in savings in save-or-spend dilemmas. Finally, Experiment 4 that was carried out on an American (as opposed to Polish) sample provided some evidence for the cross-cultural stability of the symbolic function of saving as a buffer of death anxiety.

Taken together, our findings present a consistent picture and suggest that saving money can be a potent buffer against death anxiety. This conclusion is particularly reinforced by the results of Experiment 1, which assessed in an unusually direct way the hypothesis that saving mollifies death anxiety. Typically, in terror management research participants are primed with mortality salience and, then, the level of the hypothesized anxiety buffering variable (e.g., saving intentions) is compared to a control condition. Any observed difference is considered to support the existentially buffering role of the hypothesized variable. While this approach is extremely valuable (indeed, the remaining experiments we report in this paper rely on that same experimental design), in Experiment 1 we applied a reverse approach: Participants were primed with spending or saving money and, then, asked to declare their fear of death. The results obtained in this way–that thinking about spending or saving reduces reported death fear–were much more direct and unambiguous as to the existentially soothing role that saving plays.

The results presented in this paper not only reaffirmed previous findings that consumption protects people from existential anxiety (see e.g., [10], [38]), but revealed furthermore that saving can be an even more effective buffer than buying in soothing fear of death. Earlier studies examining consumption in the context of the terror management theory framework had exclusively focused on spending-related scenarios: Participants were asked to rate advertisements of luxury products [10] or to indicate the amount they expected to spend on luxury items [34]. Yet they were never forced to make a choice between spending now or saving for the future. Using an experimental design, in which participants had to choose between spending and saving, however, we were able to demonstrate that when it comes to dealing with death thoughts, saving seems to be more effective than spending.

Our results, in one sense, are counterintuitive. One might have reasonably expected that mortality salience, with its suggestion of the shortness and uncertainty of life, could have prompted participants to spend, to shift to a “let us eat and drink; for tomorrow we shall die” mentality. However, instead of shortsightedness, primes of mortality have induced a willingness to invest in the future. Why does saving shield against existential anxiety, and why is it a more effective shield than consumption? Having a nest egg to face unexpected and potentially large expenses can provide a sense of security against life’s vicissitudes and lead to a more optimistic outlook in life. In this sense, saving provides a more direct, instrumental way of coping with the fear of death. At the same time, being a successful saver can endow a person with feelings of control, competence, and self-worth, all of which can effectively buffer existential anxiety in a more symbolic or indirect way.

Conspicuous consumption can enhance self-esteem and procure social validation as well, at least for a temporary time. Yet it cannot lighten the anxiety born from uncertainty, or provide a sense of mastery over one’s life. On the contrary, reckless consumption over time can lead to substantial financial troubles, which would surely aggravate future related anxieties and drain one’s self-esteem. Maheswaran and Agrewal [40] propose that mortality salience induces both a desire to attain social acceptability (i.e., the impression motivation) and a desire to defend one’s worldview (i.e., the defense motivation). Conspicuous consumption is undoubtedly a means to impress others, while saving, which is a responsible and rational financial behavior, may be both a way to live up to one’s culture’s values and impress others. As a whole then, it seems that saving harbors a greater existential protection potential than consumption does, which explains our findings.

The results of the present studies echo earlier findings on the effects of mortality salience on money and consumption. Accumulation of wealth provides security–both instrumental and symbolic. As reviewed earlier, wealth and possessions function to buffer existential anxiety–reminders of mortality increase the desire for conspicuous consumption and materialism. Whereas consuming and spending involve signaling wealth in the form of material possessions [7], [40], saving entails accumulating wealth for potential future use. On the one hand, then, saving and spending can be considered two sides of the same coin: They are both potentially capable of soothing existential anxiety, because they both imply wealth, and wealth is existentially protective in various ways.

On the other hand, in the long run, signaling wealth through consumption and possession might not be the optimal coping strategy. The age we live in is characterized by increasingly high levels of materialism and dysfunctional buying behavior (e.g., excessive buying, compulsive buying) [48], [49]. People, and particularly anxious, insecure people, resort to “retail therapy” to construct their identity through the symbolic power of material goods, to close the gap between their ideal and actual self [48], [50], [51], and ultimately, to cope with the knowledge of their mortality. Yet even though such consumption behavior can feel good in the short term and soothe existential anxiety, it appears to be a poor coping mechanism in the long term: Studies consistently show that a strong materialistic value orientation is related to lower happiness, and lower psychological and physical health [27]. Spiritual emptiness and lost connections to family, community, and nature are some of the other risks associated with materialism. In contrast, our results suggest that saving is an effective long-term existential coping strategy, because the psychological ingredients necessary for a happy life and a healthy anxiety buffer are provided by it in a more sustainable fashion. Thus, despite the general discomfort of choosing long-term over the short-term and despite the economically mandated cultural push toward consumerism, people should remember that the happiness and existential well-being rewards associated with saving far outweigh those of materialistic consumption.

A caveat about the present work is that we did not collect any truly behavioral data on saving, but relied instead on quasi-behavioral data obtained in the context of a paper-and-pencil questionnaire. In other words, we captured participants’ intentions and desired decisions about saving, but not their actual saving behavior. It is within the realm of possibility that whereas mortality salience renders the *idea* of saving more attractive, the actual *act* of saving might become more difficult in the face of death reminders. Previous research shows that mortality salience is associated with diminished self-control [52]. Self-control, in turn, is essential to saving, as it involves resisting the temptation to spend. Hence, even though saving might provide better protection from death anxiety, depleted self-control in the face of mortality thoughts could lead participants to consume more and save less. We have demonstrated that activating mortality thoughts amplifies the desire to save. Whether this desire will translate into actual saving behavior, however, might be determined by additional individual and contextual factors. Further research into these factors would be welcomed.

Future studies could also examine whether saving helps to reduce psychological defenses typically observed after mortality reminders, like expressing greater prejudice against an out-group member as a mechanism of cultural worldview defense [53]. It would also be interesting to explore the anxiety-buffering role of saving in contexts other than mortality salience. Does saving work equally well with other sources of anxiety such as uncertainty or meaninglessness, or with generalized anxiety? A related and important question concerns where a healthy, anxiety-shielding approach toward saving ceases and excessive, maladaptive saving starts. For example, the condition called hyperopia (farsightedness), where people are so obsessed with preparing for the future that they hardly enjoy the present and end up regretting their lost opportunities [54], does not sound like a desirable way of dealing with anxiety. Another case of excessive saving, hoarding of possessions [55], albeit apparently driven by anxiety, does not represent healthy coping with anxiety either. We thus wish to emphasize that there are boundaries to the anxiety-buffering capacity of saving.

## Conclusion

People’s decisions to save or spend money have important implications on both individual and societal levels. A high level of household savings is beneficial to a nation’s economy (e.g., [56]), and in a world struggling with depletion of resources and scarcity, thriftiness and putting a break on excessive consumption seem to be laudable goals. On an individual level, saving money is associated with financial well-being, which has the potential to significantly affect one’s overall well-being. Our results suggest that, additionally, saving money also plays an important psychological role as a buffer against death anxiety, and is probably a sturdier buffer than consumption. People who wish to boost their existential well-being might thus be better off choosing saving over spending.

## References

[1] Maslow AH (1943) A theory of human motivation. Psychological Review 50: 370–396.

[2] Furnham A, Argyle M (1998) The psychology of money. London: Routledge.

[3] Bakshi GS, Chen Z (1996) The spirit of capitalism and stock-market prices. The American Economic Review 86: 133–157.

[4] Belk RW (1988) Possessions and the extended self. Journal of Consumer Research 15: 139–168.

[5] Kasser T, Ryan RM, Couchman CE, Sheldon KM (2004) Materialistic values: Their causes and consequences. In: Kasser T, Kanner AD, editors. Psychology and Consumer Culture. Washington, DC: American Psychological Association. 11–28.

[6] Griskevicius V, Tybur JM, Sundie JM (2007) Blatant benevolence and conspicuous consumption: When romantic motives elicit strategic costly signals. Journal of Personality and Social Psychology 93: 85–102 10.1037/0022-3514.93.1.85 17605591

[7] Sundie JM, Kenrick DT, Griskevicius V, Tybur JM, Vohs KD, et al (2011) Peacocks, Porsches, and Thorstein Veblen: Conspicuous consumption as a sexual signaling system. Journal of Personality and Social Psychology 100: 664–680 10.1037/a0021669 21038972

[8] Stillman TF, Fincham FD, Vohs KD, Lambert NM, Phillips CA (2012) The material and immaterial in conflict: Spirituality reduces conspicuous consumption. Journal of Economic Psychology 33: 1–7 10.1016/j.joep.2011.08.012

[9] Pyszczynski T, Greenberg J, Solomon S, Koole SL (2010) Experimental existential psychology: Coping with the facts of life. In: Fiske ST, Gilbert DT, Lindzey G, editors. Handbook of Social Psychology. Hoboken, NJ: Wiley. 724–57.

[10] Mandel N, Heine S (1999) Terror management and marketing: He who dies with the most toys wins. In: Arnould EJ, Scott LM, editors. Advances in Consumer Research 26: 527–532. Provo, UT: Association for Consumer Research.

[11] Zaleskiewicz T, Gasiorowska A, Kesebir P, Luszczynska A, Pyszczynski T (2013) Money and the fear of death: The symbolic power of money as an existential anxiety buffer. Journal of Economic Psychology 36: 55–67 10.1016/j.joep.2013.02.008

[12] Kesebir P, Pyszczynski T (2012) The role of death in life: Existential aspects of human motivation. In: Ryan RM, editor. The Oxford Handbook of Human Motivation. New York, NY: Oxford University Press. 43–64.

[13] Greenberg J, Solomon S, Pyszczynski T (1997) Terror management theory of self-esteem and social behavior: Empirical assessments and conceptual refinements. In: Zanna MEP, editor. Advances in Experimental Social Psychology 29: 61–139. New York, NY: Academic Press.

[14] Solomon S, Greenberg J, Pyszczynski T (1991) A terror management theory of social behavior: The psychological functions of self-esteem and cultural worldviews. In: Zanna MEP, editor. Advances in Experimental Social Psychology 24: 93–159. San Diego, CA: Academic Press.

[15] Burke BL, Martens A, Faucher EH (2010) Two decades of terror management theory: A meta-analysis of mortality salience research. Personality and Social Psychology Review 14: 155–195 10.1177/1088868309352321 20097885

[16] Greenberg J, Pyszczynski T, Solomon S, Rosenblatt A, Veeder M, et al (1990) Evidence for terror management II: The effects of mortality salience on reactions to those who threaten or bolster the cultural worldview. Journal of Personality and Social Psychology 58: 308–318 10.1037/0022-3514.58.2.308

[17] McGregor HA, Lieberman JD, Greenberg J, Solomon S, Arndt J, et al (1998) Terror management and aggression: Evidence that mortality salience motivates aggression against worldview-threatening others. Journal of Personality and Social Psychology 74: 590–605 10.1037/0022-3514.74.3.590 9523407

[18] Rosenblatt A, Greenberg J, Solomon S, Pyszczynski T, Lyon D (1989) Evidence for terror management theory: I. The effects of mortality salience on reactions to those who violate or uphold cultural values. Journal of Personality and Social Psychology 57: 681–690 10.1037/0022-3514.57.4.681 2795438

[19] Schmeichel BJ, Gailliot MT, Filardo EA, McGregor I, Gitter S, et al (2009) Terror management theory and self-esteem revisited: The roles of implicit and explicit self-esteem in mortality salience effects. Journal of Personality and Social Psychology 96: 1077–1087 10.1037/a0015091 19379037

[20] Dechesne M, Greenberg J, Arndt J, Schimel J (2000) Terror management and the vicissitudes of sports fan affiliation: the effects of mortality salience on optimism and fan identification. European Journal of Social Psychology 30: 813–835 doi:;10.1002/1099-0992(200011/12)30:6<813::AID-EJSP17>3.0.CO;2-M

[21] Hirschberger G, Florian V, Mikulincer M (2002) The anxiety-buffering function of close relationships: mortality salience effects on the readiness to compromise mate selection standards. European Journal of Social Psychology 32: 609–625 10.1002/ejsp.110

[22] Hayes J, Schimel J, Arndt J, Faucher EH (2010) A theoretical and empirical review of the death-thought accessibility concept in terror management research. Psychological Bulletin 136: 699–739 10.1037/a0020524 20804234

[23] Harmon-Jones E, Simon L, Greenberg J, Pyszczynski T, Solomon S, et al (1997) Terror management theory and self-esteem: Evidence that increased self-esteem reduces MS effects. Journal of Personality and Social Psychology 72: 24–36 10.1037/0022-3514.72.1.24 9008372

[24] Schimel J, Hayes J, Williams T, Jahrig T (2007) Is death really the worm at the core? Converging evidence that worldview threat increases death-thought accessibility. Journal of Personality and Social Psychology 92: 789–803 10.1037/0022-3514.92.5.789 17484605

[25] Diener E, Biswas-Diener R (2008) Happiness: Unlocking the mysteries of psychological wealth. New York, NY: Wiley-Blackwell.

[26] Dittmar H (2010) Consumer culture, identity and well-being: The search for the 'good life' and the 'body perfect'. New York, NY: Psychology Press.

[27] Kasser T (2003) The high price of materialism. Cambridge, MA: The MIT Press.

[28] Fromm E (1976) To have or to be?. New York, NY: Harper and Row.

[29] Hirschman EC (1990) Secular immortality and the American ideology of affluence. Journal of Consumer Research 17: 31–42.

[30] Maslow AH (1954) Motivation and personality. New York, NY: Harper and Row.

[31] Yalom ID (1980) Existential psychotherapy. New York, NY: Basic Books.

[32] Arndt J, Solomon S, Kasser T, Sheldon KM (2004) The urge to splurge: A terror management account of materialism and consumer behavior. Journal of Consumer Psychology 14: 198–212 10.1207/s15327663jcp14032

[33] Solomon S, Greenberg J, Pyszczynski T (2004) Lethal consumption: Death-denying materialism. In: Kasser T, Kanner AD, editors. Psychology and Consumer Culture. Washington, DC: American Psychological Association. 127–146.

[34] Kasser T, Sheldon KM (2000) Of wealth and death: Materialism, mortality salience, and consumption behavior. Psychological Science 11: 348–51 10.1111/1467-9280.00269 11273398

[35] Rindfleisch A, Burroughs JE, Wong N (2009) The safety of objects: Materialism, existential insecurity, and brand connection. Journal of Consumer Research 36: 1–16 10.1086/595718

[36] Lunt PK, Livingstone SM (1991) Psychological, social and economic determinants of saving: Comparing recurrent and total savings. Journal of Economic Psychology 12: 621–641 10.1016/0167-4870(91)90003-C

[37] Wärneryd KE (1989) On the psychology of saving: An essay on economic behavior. Journal of Economic Psychology 10: 515–541 10.1016/0167-4870(89)90041-X

[38] TNS Poland (2012) Postawy Polaków wobec oszczędania. Kronenberg Foundation Report. Available: http://www.citibank.com/poland/kronenberg/polish/files/fk_oszcz_2012.pdf. Accessed 2013 September 18.

[39] The Futures Company (2009). Understanding consumer attitudes to saving 2004–2008. Aviva Report. Available: www.aviva.com/media/upload/ucas_survey.pdf. Accessed 2013 September 18.

[40] Maheswaran D, Agrawal N (2004) Motivational and cultural variations in mortality salience effects: Contemplations on terror management theory and consumer behavior. Journal of Consumer Psychology 14: 213–218 10.1207/s15327663jcp14033

[41] Ryan RM, Deci EL (2000) Self-determination theory and the facilitation of intrinsic motivation, social development, and well-being. American Psychologist 55: 68–78. 11392867 10.1037//0003-066x.55.1.68

[42] Kasser T (2011) Can thrift bring well-being? A review of the research and a tentative theory. Social and Personality Psychology Compass 5: 865–877.

[43] Dittmar H, Bond R, Hurst M, Kasser T (2013) A meta-analysis of the materialism literature. Unpublished manuscript, University of Sussex, Brighton, UK.

[44] Liu W, Aaker J (2007) Do you look to the future or focus on today? The impact of life experience on intertemporal decisions. Organizational Behavior and Human Decision Processes 102: 212–225 10.1016/j.obhdp.2006.02.004

[45] Watson D, Clark LA, Tellegen A (1988) Development and validation of brief measures of Positive and Negative Affect: The PANAS scales. Journal of Personality and Social Psychology 54: 1063–1070 10.1037/0022-3514.54.6.1063 3397865

[46] Templer DI (1970) The construction and validation of a death anxiety scale. Journal of General Psychology 82: 165–177 10.1080/00221309.1970.9920634 4394812

[47] Pyszczynski T, Greenberg J, Solomon S (1999) A dual-process model of defense against conscious and unconscious death-related thoughts: An extension of terror management theory. Psychological Review 106: 835–45 10.1037/0033-295X.106.4.835 10560330

[48] Dittmar H (2011) Material and consumer identities. In: Schwartz SJ, Luycks K, Vignoles VL, editors. Handbook of Identity Theory and Research. New York: Springer. 745–769.

[49] Pryor JH, Hurtado S, Saenz VB, Santos JL, Korn WS (2007) The American freshman: Forty year trends. Los Angeles: Higher Education Research Institute.

[50] Dittmar H, Bond R (2010) I want it and I want it now: Self-discrepancies and materialistic values as predictors of ordinary and compulsive buyers’ temporal discounting of different consumer goods. British Journal of Psychology 101: 751–776. 20128957 10.1348/000712609X484658

[51] Dittmar H, Kapur P (2011) Consumerism and well-being in India and the UK: Identity projection and emotion regulation as underlying psychological process. Psychological Processes: 56, 71–85.

[52] Gailliot MT, Schmeichel BJ, Baumeister RF (2006) Self-regulatory processes defend against the threat of death: Effects of self-control depletion and trait self-control on thoughts and fears of dying. Journal of Personality and Social Psychology 91: 49–62 10.1037/0022-3514.91.1.49 16834479

[53] Greenberg J, Pyszczynski T, Solomon S, Simon L, Breus M (1994) Role of consciousness and accessibility of death-related thoughts in mortality salience effects. Journal of Personality and Social Psychology 67: 627–637. 7965609 10.1037//0022-3514.67.4.627

[54] Kivetz R, Keinan A (2006) Repenting hyperopia: An analysis of self-control regrets. Journal of Consumer Research 33: 273–282.

[55] Frost RO, Gross RC (1993) The hoarding of possessions. Behaviour Research and Therapy 31: 367–381. 8512538 10.1016/0005-7967(93)90094-b

[56] Lea SEG, Tarpy RM, Webley P (1987) The individual in the economy: A survey of economic psychology. Cambridge: Cambridge University Press.

